# A New Derivatization Reagent for HPLC–MS Analysis of Biological Organic Acids

**DOI:** 10.1007/s10337-017-3421-0

**Published:** 2017-10-29

**Authors:** Bryce J. Marquis, Hayley P. Louks, Chhanda Bose, Robert R. Wolfe, Sharda P. Singh

**Affiliations:** 1 Department of Geriatrics, University of Arkansas for Medical Sciences, Little Rock, AR USA; 2 University of Central Arkansas, Conway, AR USA; 3 Department of Pharmacology and Toxicology, University of Arkansas for Medical Sciences, Little Rock, AR USA

**Keywords:** LC MS/MS, Derivatization, TCA cycle intermediates, Krebs cycle, Metabolomics, 4-BNMA

## Abstract

Small molecules containing carboxylic acid functional groups are ubiquitous throughout biology, playing vital roles in biological chemistry ranging from energy metabolism to cellular signaling. This paper describes a new derivatization reagent, 4-bromo-*N*-methylbenzylamine, which was selected for its potential to derivatize mono-, di- and tri-carboxylic acids, such as the intermediates of the tricarboxylic acid (TCA) cycle. This derivatization procedure facilitated the use of positive electrospray ionization (ESI) tandem mass spectrometry (MS/MS) detection of derivatized species allowing for clear identification thanks to the easily recognizable isotope pattern of the incorporated bromine. A liquid chromatography (LC)-MS/MS method was developed which provided limits of detection between 0.2 and 44 μg L^−1^ in under 6 min, depending on the analyte and total analysis time. This method was successfully applied in both in vitro and in vivo models.

**Electronic supplementary material:**

The online version of this article (doi:10.1007/s10337-017-3421-0) contains supplementary material, which is available to authorized users.

## Introduction

Many biologically important small molecules contain carboxylic acid functional groups; these molecules are particularly relevant in central carbon energy metabolism [e.g., in the tricarboxylic acid (TCA) cycle]. Metabolic analysis relies heavily on liquid chromatography mass spectrometry (LC–MS) applications that frequently employ tandem mass spectrometry (MS/MS), which benefits from increased sensitivity and specificity using multiple reaction monitoring (MRM) methods [1–3]. Short chain carboxylic acids suffer from poor retention using traditional reverse phase LC methods and poor sensitivity due to reduced ionization efficiency and increased ion suppression using the MS in electrospray ionization (ESI) [4]. A variety of approaches are employed to improve LC–MS analysis of these compounds, including the use of ion pairing agents [5–8] or hydrophilic interaction chromatography based column separations [9–13].

Chemical derivatization techniques pose a promising approach to overcoming these challenges because they offer the opportunity to increase analytical sensitivity and chromatographic retention through the selection of a derivatization reagent with desirable physical and chemical properties [14–17]. Activating carboxylic acids with a carbodiimide agent [such as 1-ethyl-3-dimethylaminopropyl carbodiimide (EDC)] for amide coupling provides a favorable platform for derivatization of small biologically relevant carboxylic acids [18–21] since the activation can be performed rapidly under mild, aqueous conditions. EDC-mediated coupling of biological carboxylic acids with an array of amines with different properties has been explored to tailor the properties of the derived product for LC–MS applications. For example, EDC coupling with (2-(4-aminophenoxy)ethyl)(4-bromophenethyl)-dimethylammonium bromide (4-APEBA) was used to improve the sensitivity of LC–MS/MS detection of carboxylic acids due to the permanent positive charge of the amide on 4-APEBA [20]. In addition, the isotopic pattern of the 4-APEBA incorporated bromide provided highly specific recognition of derived compounds. However, 4-APEBA coupling of species containing multiple carboxylic acids (such as TCA cycle intermediates) is non-ideal due to the potential for multiple end products because of internal cyclization reactions and electrostatic repulsion between 4-APEBA and intermediate derived productions.

To overcome the derivatization challenges arising from the multiple reactive carboxylic acid sites of TCA cycle intermediates, another group selected a reagent (O-benzylhydroxylamine [O-BHA]) with an amine pKa value of 4.8, which is low enough to ensure that the amine is uninhibited by protonation in ideal EDC reaction pH ranges, facilitating improved reaction kinetics which prevent unwanted internal or incomplete reactions [21]. In another approach, an *N*-methlylated amine (*N*-methyl-2-phenylethanamine [MPEA]) was used successfully for sensitive LC–MS/MS analysis of TCA intermediates [20]. Since MPEA is a secondary amine, no undesirable internal reactions could occur, and consistently complete labeling of the TCA intermediates was found.

In this article, we describe an alternative derivatization strategy that combines favorable characteristics of other EDC amine carboxylic acid coupling approaches using 4-bromo-*N*-methylbenzylamine (4-BNMA) as a derivatization reagent for TCA intermediates and other biologically important carboxylic acids. We chose 4-BNMA for the following reasons:

1. It has a secondary amine which prevents unwanted internal cyclization reactions and facilitates complete derivatization of small organic polyacids.
2. It features a phenyl group which improves retention in reversed phase chromatography and offers favorable fragmentation for tandem MS approaches.
3. It incorporates a bromine in the derivatized product which allows for improved identification due to the isotope pattern of bromine.
4. Bromine’s isotope pattern can provide insight into the identification of unknown species, since the addition of multiple 4-BNMA molecules provides a pattern that can inform how many carboxylic acids groups are present on the species in question (Fig. 1).

**Fig. 1:**
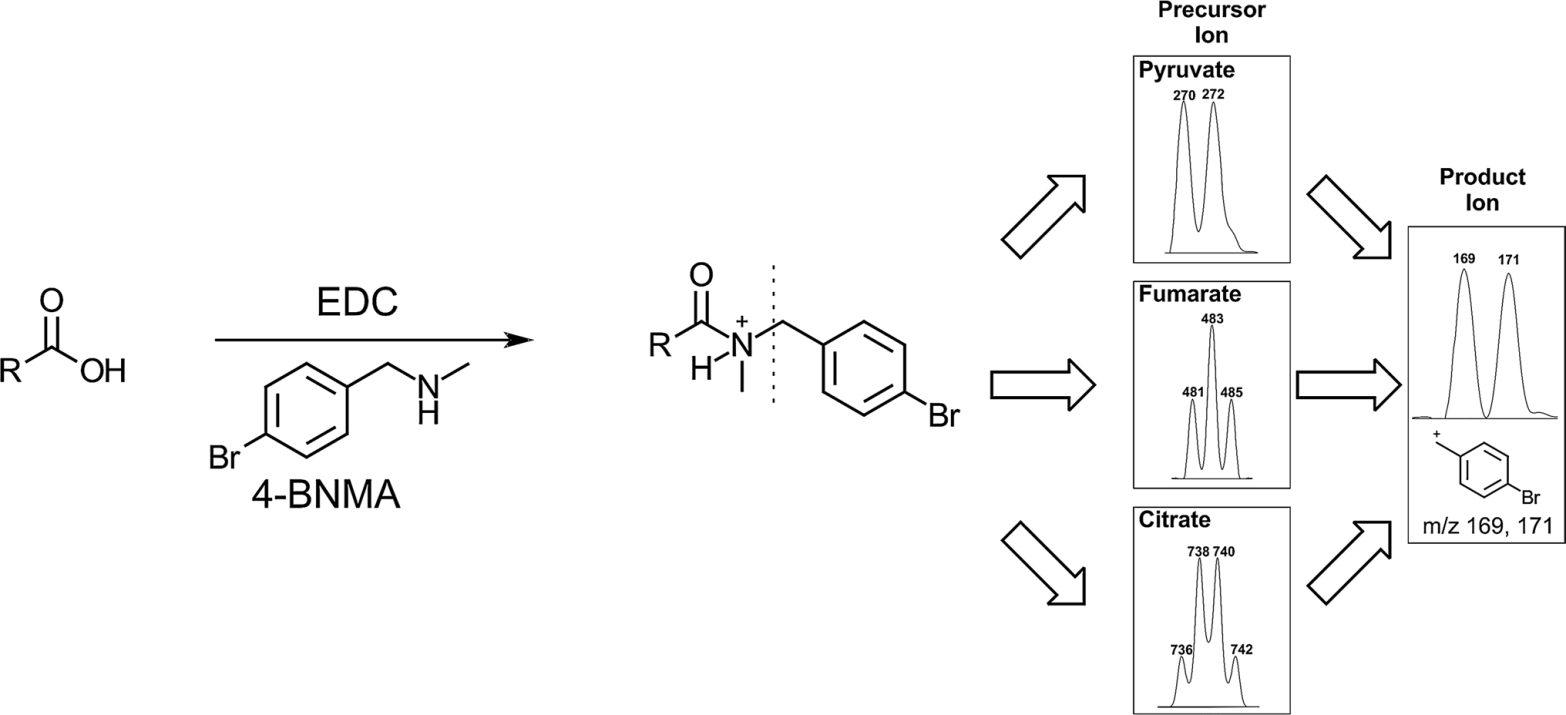
Overview of 4-BNMA derivatization scheme. Simplified 4-BNMA derivatization of generic carboxylic acid is shown on the left. Dotted line represents location of fragmentation in MRM analysis. The right depicts the ESI + precursor M + H isotope patterns for representative doublet from a mono-(pyruvate), triplet from a di-(fumarate) and quartet from a tri-(citrate) 4-BNMA derivatized carboxylic acid and the isotope pattern from the highest intensity product ion signal resulting from a resonance stabilized ion fragment from 4-BNMA
5. It is widely commercially available and inexpensive, making it easily accessible.

## Materials and Methods

### Reagents

Acetic acid, lactic acid (LA), pyruvic acid (PA), citric acid (CA), isocitric acid (ICA), succinic acid (SA), 2-hydroxy-glutaric acid (2-OHGA), a ketoglutaric acid (αKG), oxaloacetic acid (OA), malic acid (MA), fumaric acid (FA), methyl malonic acid (MMA), maleic acid (MAEA) and formic acid were all purchased from Sigma Aldrich (Milwaukee, WI) in the highest purity available. 1-Ethyl-3-dimethylaminopropyl carbodiimide (EDC) was purchased from Acros Organics (Thermo Fisher Scientific, Waltham, MA). HPLC-grade acetonitrile and methanol were purchased from Sigma Aldrich, and water was purified using in house Milli-Q water purification system. Brominated derivatization agents 4-BNMA, 3-methyl-*N*-methylbenzylamine (3-BNMA), and 4-bromo-*n*-methylaniline (4-BMA) were purchased from Sigma Aldrich and 2-(4-Bromophenyl)-*N*-methylethanamine (B-MPEA) was purchased from Combi-locks Inc. (San Diego, CA). Isotope standards sodium lactate 13C_3_ (LAi), citric D_4_(2,2,4,4) acid (CAi), fumaric ^13^C_4_ acid (FAi), sodium pyruvate ^13^C_3_ (PAi), and α-ketoglutaric D_6_ acid (αKGi) were purchased from Isotec (Sigma Aldrich, Milwaukee, WI) and succinic D_6_ acid (SAi), and disodium 2 hydroxy glutarate D_3_(2,3,3) (2OHGAi) were purchased from CDN Isotopes (Pointe-Claire, Canada).

### Equipment

#### Sample Preparation Equipment

Homogenization of tissue and cell culture samples was conducted using a Beadruptor 24 (Omni International Inc., Kennesaw, GA). Tissue samples were homogenized in stainless steel tubes loaded with silicon carbide sharp particles, both purchased from Biospec Products (Bartlesville, OK), and cultured cells were homogenized in 0.5 mL tubes loaded with 1.4 mm ceramic beads. Centrifugation was conducted in a Thermo Legend Micro 21R and ample drying was conducted using a Thermo Savant Speedvac Plus (Waltham, MA).

#### Chromatography Mass Spectrometry

All LC–MS/MS was conducted using an ABSciex Qtrap 5500 mass spectrometer with an Eksigent Micro 200 LC and was controlled by Analyst 1.6.3 software (Sciex Inc., Framingham, MA). Separations were conducted using 15 cm ChromXP-C18EP column with 3 micron particles and 300 A pore size (Sciex Inc.). Mobile phases consisted of MilliQ water with 1% formic acid (*A*) and acetonitrile (ACN) with 1% formic acid (*B*). A 50:50 (*A*:*B*) isocratic elution program was used for the initial reaction optimization experiments in which a single analyte and internal standard were measured with a 25 μL min^−1^ flow rate. Five microliter injections of samples from mixtures of multiple standards, tissue samples, and cultured cell samples were resolved using a gradient elution program ran at 25 μL min^−1^ with the initial conditions held at 40% B for 30 s, then a linear gradient to 95% B for 5 min and held for an additional 30 s, followed by a 1 min post conditioning at 100% B. Total analysis time was 7 min. A three-port valve was used to direct the eluent towards waste during the first 30 s and last minute, while the middle 5.5 min it directed the eluent toward the mass spectrometer to avoid contamination of the ionization source.

MS detection was conducted using an ABSciex Qtrapp 5500 equipped with a Turbo V ion source with a 50 micron ESI probe electrode operated at unit resolution. The source was operated at positive 5.5 kV and a temperature of 450^o^ C. The MS declustering potential was maintained at 110 V, entrance potential of 10 V, and a collision exit potential of 16 V, and curtain gas was 20. Multiple reaction monitoring (MRM) parameters were optimized using direct injection of 50 mg L^−1^ standards using a syringe pump operated at 10 μL min^−1^ using automated compound optimization controlled by Analyst 1.6.3 software. The final programs used optimized MRM parameters with 20 ms hold times in the order described in Table 1. Peak areas for analytes and standards were measured using the MultiQuant package in Analyst 1.6.3 and statistical analysis was conducted using R software.

**Table 1:** Summary of LC MS/MS detection settings and characteristics for biologically important organic acids determined by the 4-BNMA derivatization method

Analyte	MRM transitions	RT (min)	CE	IS	LOD (μg L^−1^)	LOQ (μg L^−1^)	Matrix effect (%)	Inter-day RSD (%)	Intra-day RSD (%)
LA	**272/169**	1.95	35	LAi	10	30	107	4	4
	274/171								
PA	**270/169**	2.65	37	Pai	5	15	94	4	6
	272/171								
MA	**497/169**	3.4	51	SAi	4	12	–	1	4
	499/169								
	499/171								
	501/171								
FA	**479/169**	3.7	41	FAi	0.9	3	95	1	4
	481/169								
	481/171								
	483/171								
MAEA	**479/169**	3.7	47	FAi	0.4	1	–	3	5
	481/169								
	481/171								
	483/171								
SA	**481/169**	3.85	57	SAi	0.3	1	100	0.5	4
	483/169								
	483/171								
	485/171								
MMA	**481/169**	3.85	50	SAi	2	6	–	4	5
	483/169								
	483/171								
	485/171								
αKG	**509/169**	4.1	45	αKGi	3	9	95	2	6
	511/169								
	511/171								
	513/171								
OA	**495/169**	4.3	50	αKGi	49	148	–	2	5
	497/169								
	497/171								
	499/171								
2OHGA	**511/169**	4.7	57	2OHGAi	30	91	104	5	6
	513/169								
	513/171								
	515/171								
ICA	**736/169**	4.65	57	CAi	2	6	–	5	6
	738/169								
	738/171								
	740/169								
	740/171								
	742/171								
CA	**736/169**	5.25	57	CAi	2	6	94	4	4
	738/169								
	738/171								
	740/169								
	740/171								
	742/171								

MRM transitions used for calibration and determination of in vivo and in vitro sample concentrations is bolded. LOD and LOQ were determined for aqueous standards and matrix effect was determined with porcine skeletal muscle extract as matrix. Inter-day RSD and intra-day RSD were determined by averaging triplicate measurements of aqueous standards at three different concentrations

### Experimental

#### Selection of Derivatization Reagent

The protocol described by Kloos et al. optimized for MPEA [20] was used as the basis for testing EDC coupling of succinic acid (SA) with a series of brominated, *N*-methylated amine derivatization reagents. Briefly, 15 μL of serial dilution generated succinic acid standards in water were mixed with 50 μL of the derivatization reagents which were prepared 10 mM in ACN, following which 25 μL of freshly made 1 M EDC in ACN-H_2_O (90:10; v/v) were added, samples were mixed and then held at 60 °C for 45 min. Samples were dried under speedvac and reconstituted in 200 μL ACN-H_2_O (50:50; v/v). MRM optimization was conducted by direct infusion into the MS at 10 μl/min and the limits of detection (LOD) and limits of quantitation (LOQ) were determined by HPLC MS using the isocratic method described in the previous section. LOD and LOQ were determined following FDA’s guidelines using the ratio of either 10 (LOQ) or 3.3 (LOD) times the standard deviation of a low concentration standard and the slope of the calibration response [22].

#### Optimization of Derivatization Reagent Concentration, Temperature, and Reaction Time

To optimize the reaction time and temperature, a series of derivatizations of 12.5 μl aliquots of 50 μg L^−1^ succinic acid samples were performed at either room temperature (approximately 22 °C), 37 °C, and 60 °C. Samples were derivatized with the addition of 50 μL of 10 mM 4-BNMA followed by 25 μL of 1 M EDC and the reaction was allowed to proceed for up to 4 h. Samples designated for different reaction times (0, 5, 10, 15, 30, 45, 60, 90, 120, 240 min) were quenched through the addition of 100 μL of 50 mM acetate buffer (pH 5.6). Simultaneously, a sample of 50 μg L^−1^ SAi was derivatized for the entire 4 h prior to quenching with acetate. Each of the SA samples was mixed with an equal volume of SAi then the samples were dried by speedvac, reconstituted in ACN-H_2_O (50:50; v/v) and ran on an isocratic HPLC MS method. The ratio of the peak areas of SA/SAi were used to determine the reaction completion. 4-BNMA concentration was optimized by derivatizing 12.5 μl 50 μg L^−1^ SA samples with 50 μL of either 1, 10 or 100 mM 4-BNMA followed by 25 μL of 1 M EDC and allowed to react for 45 min at 60 °C.

#### Stability Tests

To determine the long-term stability of derivatized compounds for storage and future analysis, a series of experiments were conducted to compare previously prepared 4-BNMA SA derivatives with freshly prepared 4-BNMA SAi derivatives. SA experimental samples were derivatized using 4-BNMA as described above (12 μL of 50 μg L^−1^ SA), dried by speedvac and then stored at room temperature, 4, − 20 and − 80 °C. After the allotted time (6, 12, 24, 48, 72 h, 1, 2 weeks, 1, 6 months), a 12.5 μl aliquots of 50 μg L^−1^ solution of SAi was derivatized with 4-BNMA as described above, dried and then reconstituted in ACN-H_2_O (50:50; v/v). Then the reconstituted SAi derivatized solution was transferred to an experimental SA sample of the allotted time in order reconstitute the SA derivative. The mixture was run using the isocratic HPLC MS program and the ratio of the peak areas of SA/SAi were used to determine the stability.

#### Optimization of LC Parameters, Limits of Detection, and Precision Determination

**Fig. 2:**
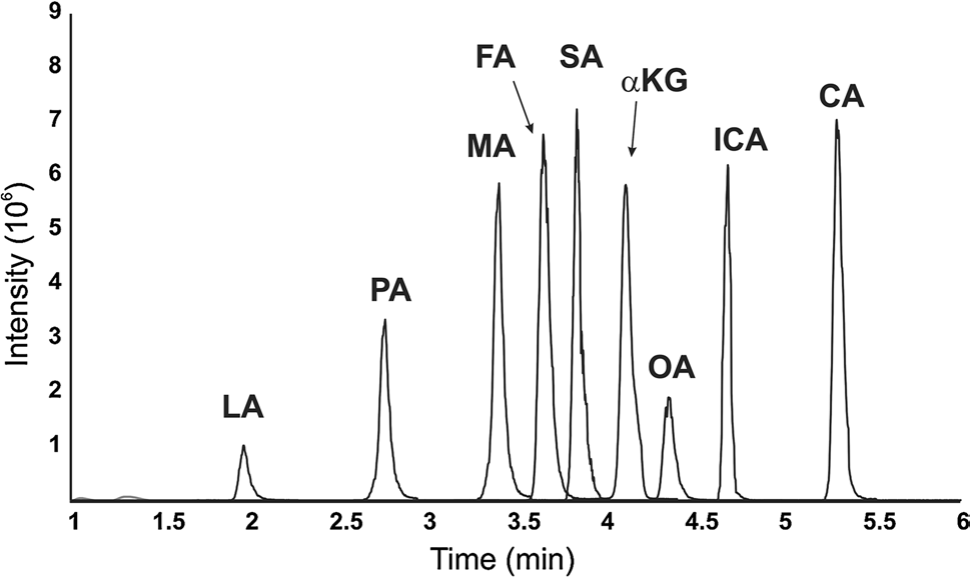
Chromatogram of TCA intermediates. Combined extracted ion chromatogram of MRMs (see Table 1) illustrating the elution order of 4-BNMA derivatized TCA intermediate aqueous standards at either 100 μg L^−1^ (MA, FA, SA, aKG, ICA, CA) or 250 μg L^−1^ (LA, PA, OA)

Gradient programs were optimized maximizing the peak resolution between 4-BNMA SA and FA derivatives using H_2_O with 0.1% formic acid as the A phase and ACN with 0.1% formic acid as the B phase see example chromatogram of TCA intermediates in Fig. 2. Limits of detection were determined by derivatizing a series of nine standards of each analyte ranging in concentration between 50 ng L^−1^ and 500 μg L^−1^ and analyzing using the previously determined MRM and chromatographic programs. To determine inter-day variation, standards at three different concentrations [500, 100 μg L^−1^ and either 50 μg L^−1^ (for PA, MA, FA, MAEA, SA, MMA, aKG, ICA, CA) or 250 μg L^−1^ (for LA, 2OHGA and OA)] were prepared in triplicate and derivatized as described above. Intra-day variation was determined by following the inter-day procedure on three different days. Results are summarized in Table 1.

#### Tissue Levels and Matrix Effects

To investigate the applicability of the 4-BNMA derivatization for analysis of tissue levels of organic acids, a series of experiments was conducted extracting metabolites from porcine skeletal muscle and heart tissue. Porcine samples were collected from excess tissue remaining from studies conducted at the Arkansas Children’s Nutrition Center and kindly provided by Dr. Elisabet Borsheim. Tissue homogenization was conducted using an Omni Beadrupter 24 system. Stainless steel tubes were loaded with silicon carbide beads and chilled in dry ice before 20–30 mg frozen porcine skeletal muscle or heart tissue was added followed by 20 μL of chilled MeOH-H_2_O (50:50; v/v) per mg of tissue. The samples were spiked with 3 μl of an isotope standard mix containing 1.5 μg LAi, 0.3 μg CAi, 0.5 μg FAi, 2 μg PAi, 0.2 μg SAi, 1 μg 2OHGAi and 0.2 μg αKGi. Samples were homogenized in the Beadruptor using two 30 s steps at 6.3 m/s with a dry ice sample cool down between steps. The resulting homogenate was transferred to a fresh centrifuge tube and then centrifuged at 15 kg for 10 min. The supernatant was transferred and dried under speedvac, then reconstituted and derivatized as described above. Measurements were conducted in quintuplicate and concentrations were determined using internal calibration from peaks areas of MRM chromatograms of isotope labeled standards (IS) (see Table 2 for IS MRM transitions). Concentrations were reported in μg of analyte per mg of tissue.

**Table 2:** Summary table of MRM transitions optimized for IS. Bold font indicates the MRM used for quantitative measurements

IS	MRM	IS	MRM
PAi	**273/169**	CAi	**740/169**
	275/171		742/169, 742/171
LAi	**275/169**		744/169, 744/171
	277/171		746/171
FAi	**481/169**	2OHGAi	**514/169**
	483/169, 483/171		516/169, 516/171
	483/171		518/171
SAi	**485/169**	αKGi	**513/169**
	487/169, 487/171		515/169, 515171
	489/171		517/171

To investigate the influence of skeletal muscle extraction matrix effects on the 4-BNMA derivatization method analysis we followed a modified procedure described elsewhere [23]. Since skeletal muscle extracts have a large concentration of the metabolites, we investigated these effects on isotope labeled standards LAi, PAi, FAi, SAi, αKGi, 2OHGAi and CAi. Isotope labeled standards were derivatized at 5 different concentrations: between 1 mg L^−1^ and 50 μg L^−1^ for LAi, 250–12.5 μg L^−1^ for FAi, 100–5 μg L^−1^ for PAi and 2OHGAi, and 50–2.5 μg L^−1^ for SAi, αKGi, and CAi). After derivatization, standard solutions were split evenly amongst two sets of tubes and spiked with either 50 mL of extraction medium or 50 mL of extraction medium containing the extracts from 25 mg of porcine skeletal muscle processed as described previously. After running samples by LC MS/MS, matrix effects were calculated by the following equation:

$$ {\text{Matrix effects}} = \frac{{   {\text{Peak area}}_{{{\text{Standard}} + {\text{Matrix}}}} }}{{{\text{Peak area }}_{\text{Standard}} }} \times 100 $$

#### In Vitro Studies

*Cardiac and cancer cell culture* Cardiac H9c2 (ATCC CRL-1446) clonal cell line, derived from embryonic BD1X rat heart tissue, and breast cancer cell line MAT B III (ATCC CRL-1666) were purchased from the American Type Culture Collection (ATCC; Manassas, VA). H9c2 cells were maintained in high-glucose Dulbecco’s modification of Eagle’s medium (DMEM), supplemented with 10% bovine calf serum and 1% penicillin–streptomycin solution at 37 °C with 5% CO_2_. Breast cancer cell line MAT B III, grown in McCoy’s 5a medium, supplemented with 10% fetal bovine serum, were grown in a humidified 5% CO_2_ incubator at 37° C.

*Cell treatment* Cardiac H9c2 or breast cancer cells MAT B III were plated on 100 mm tissue culture dishes overnight. After 12–16 h, cells were treated with sulforaphane (SFN), concentration ranging from 10 to 80 µM. Cells were harvested 24 h after SFN treatment and washed twice with phosphate buffered saline and pelleted by low speed centrifugation before being stored at – 80 °C.

#### In Vitro Sample Processing

Cells were divided among two tubes: one designated for citrate synthase (CS) activity determination and protein analysis, the other for metabolomics analysis. The CS cells were added to a homogenization tube loaded with 1.4 mm ceramic beads and 75 μL of CelLytitc MT Cell Lysis Reagent (Sigma, Aldrich) with protease inhibitor. The CS samples were homogenized for 1 min at 5.0 m s^−1^ on the Beadruptor. The supernatant was transferred to a fresh tube and CS activity was conducted using the Citrate Synthase Assay Kit (Sigma, Aldrich) following the manufacturer’s protocol for 96 well microplate analysis. CS reactions were performed in triplicate. For metabolomics analysis, cells were added to a homogenization tube loaded with 1.4 mm ceramic beads and 75 μl of MeOH-H_2_O (50:50; v/v)spiked with 3 μL of the isotope standard mix described in “Tissue levels and matrix effects”. Samples were homogenized for 1 min at 5.0 m s^−1^ on the Beadruptor, and the homogenate was transferred to a fresh tube and dried under speedvac. The samples were derivatized with 4-BNMA and analyzed by LC–MS/MS as described in “Optimization of derivatization reagent concentration, temperature, and reaction time”. The TCA intermediate concentrations were determined as described in “Tissue levels and matrix effects”, summed and expressed in terms of μg of analyte per U of CS activity (in μmol mL^−1^ min^−1^) as determined by the CS assay, a proxy used to normalize for mitochondria number [24].

## Results and Discussion

### Selection of Derivatization Label

**Fig. 3:**
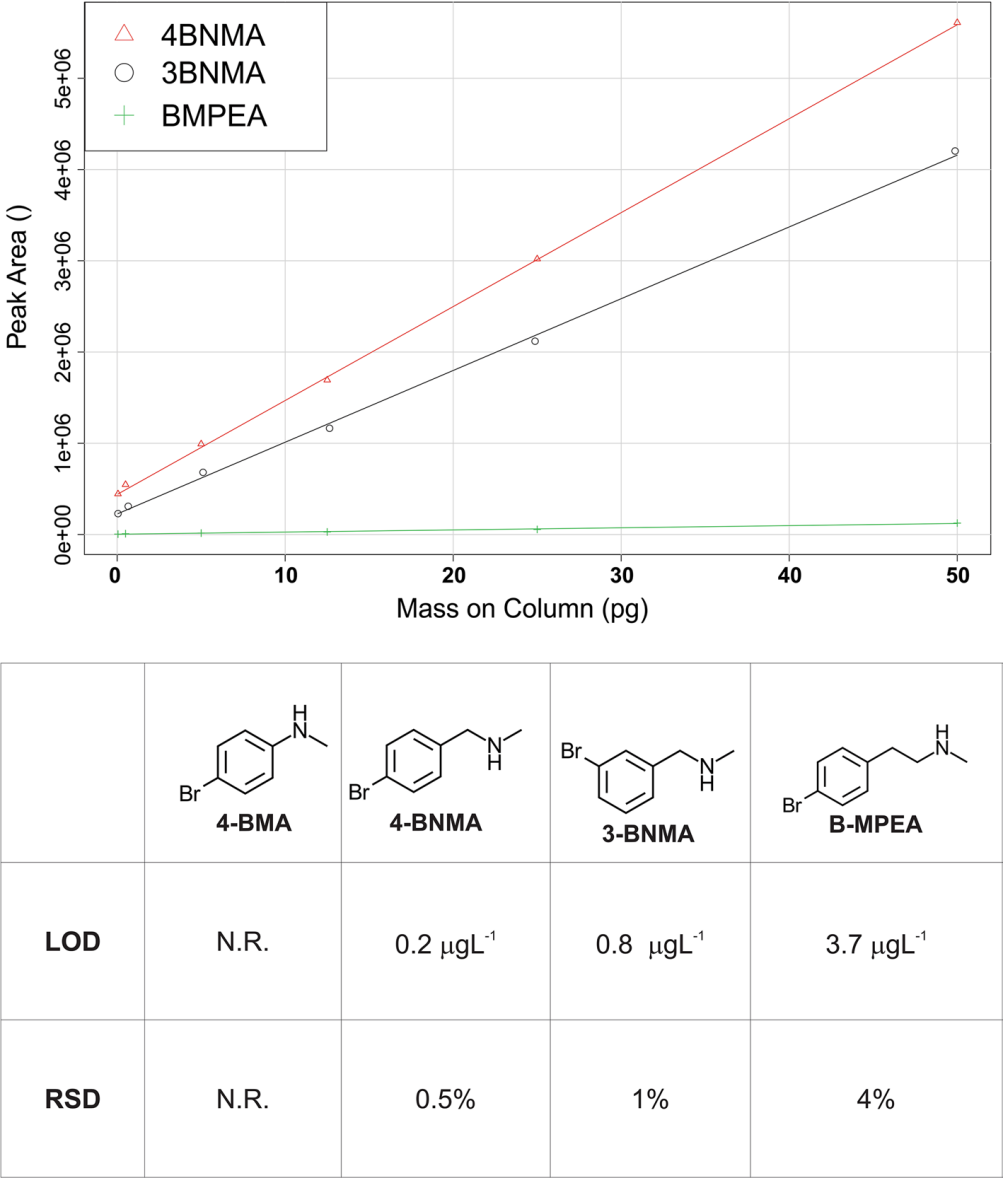
Selection of derivatization agent. Top, calibration curve arising from optimized MRM LC–MS/MS detection of succinic acid derivatives of 4-BNMA, 3-BNMA, and B-MPEA. Bottom, limits of detection and relative standard deviations for succinic acid derivatized by 4-BNMA, 3-BNMA and B-MPEA. Note that as observed elsewhere [20], we were unable to detect a derivatized product with the aniline based derivatization agent (4-BMA)

Our goal in selecting a derivatization label was to find a commercially available amine for EDC coupling that would stoichiometrically react with carboxylic acids, have favorable fragmentation characteristics for MRM detection, and incorporate a bromine to provide additional qualitative structural information. Since MPEA was the original inspiration for this work, we investigated species that were structurally similar (e.g., they featured a secondary amine and a phenyl group). We originally tested 4-BMA, 4-BNMA and B-MPEA using reaction conditions mimicking those described for MPEA [20] in a derivatization reaction with 50 μg L^−1^ succinic acid. Upon reaction completion, MRM mass spectrometry conditions were optimized for the five most prominent MRM transitions using direct infusion into the ABSciex 5500 Qtrap MS. Precursor ions were found for 4-BNMA-SA (481/483/485 *m*/*z*) and B-MPEA-SA (493/495/497 *m*/*z*) derivatives both producing strongest products ions of 169 *m*/*z* and 171 *m*/*z*. 4-BMA failed to provide a product around our expected mass in agreement with previous reports investigating the use of aniline related species as potential EDC coupling derivatization labels [20]. 4-BNMA and B-MPEA were then used to derivatize a series of SA standards between 0.1 and 500 μg L^−1^ which were then run on LC–MS/MS using isocratic elution and the previously determined MRM parameters to determine limits of detection (LOD). 4-BNMA provided the most sensitive analysis (Fig. 3). We further investigated using the meta-substituted isomer, 3-BNMA, using the same procedure but found that 4-BNMA continued to be the more desirable reagent.

### Optimization of Derivatization

**Fig. 4:**
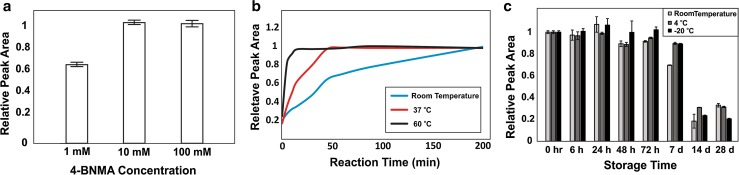
Development of 4-BNMA derivatization method for succinate. **a** Optimization of derivatization reagent concentrations. **b** Time course of 4-BNMA derivatization of succinate at different temperatures temperature. **c** Stability of 4-BNMA succinate derivatives when stored at room temperature (22 °C), 4, or − 20 °C

Label concentration, reaction time, and reaction temperature were investigated using the conditions optimized for MPEA as a starting point [20]. SA was derivatized with 4-BNMA with the label at 1, 10, and 100 mM. The optimal concentration for 4-BNMA was 10 mM, as 1 mM did not provide complete derivatization and 100 mM showed no benefit in the reaction conditions (Fig. 4). To optimize reaction time and temperature, a set of SA standards was optimized at either room temperature (22 °C), 37 °C, or 60 °C (Fig. 3). The 60 °C condition provided the fastest reaction and there were was no observed decrease in maximal intensity suggesting little thermal decomposition occurred.

### Calibration, Limits of Detection, and Matrix Effects

**Fig. 5:**
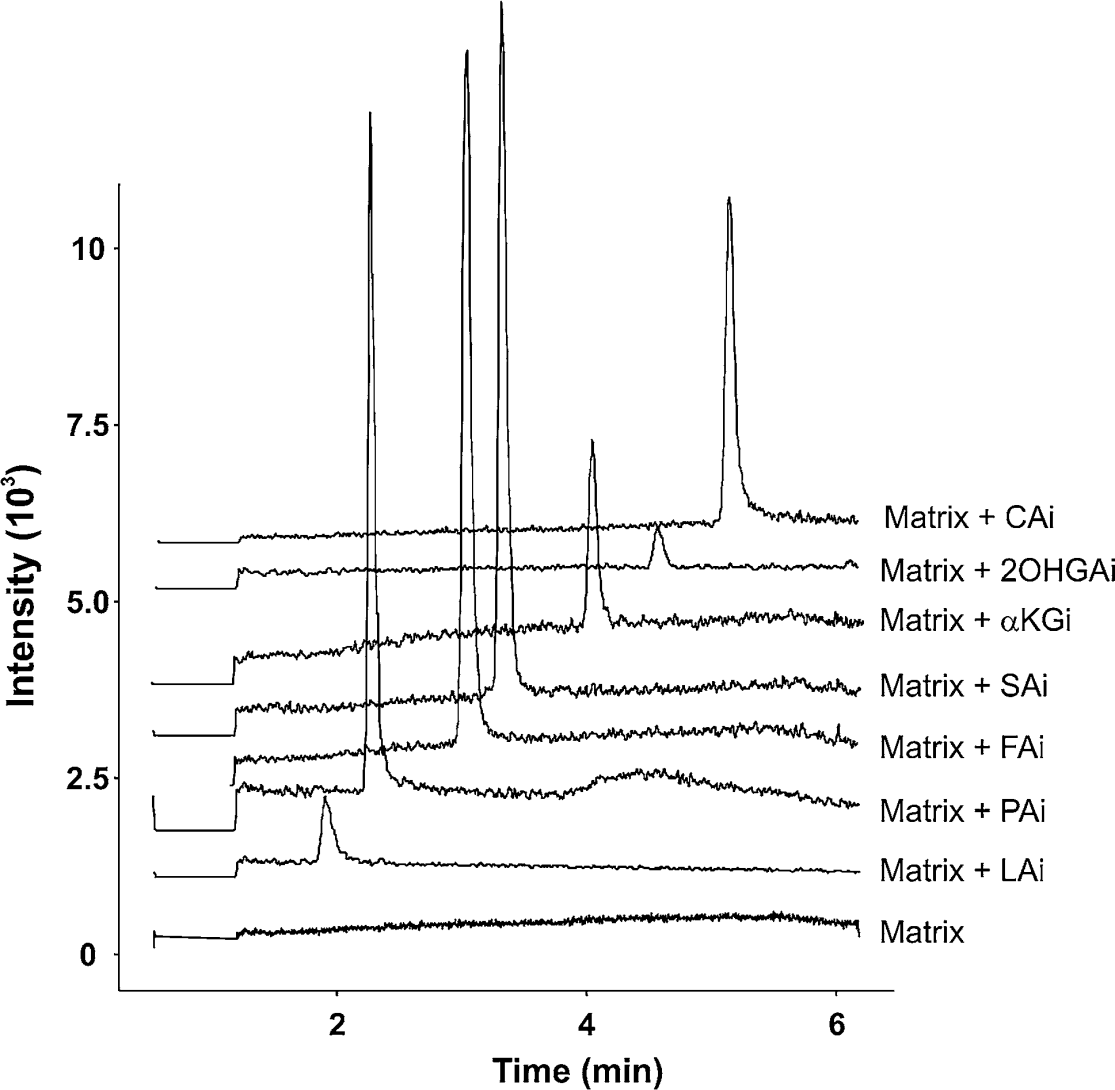
Chromatograms of unspiked and isotope labeled standard spiked porcine skeletal muscle matrix. Matrix only sample (bottom) shows total ion chromatogram from the MRM transitions for isotope labeled TCA intermediates (see Table 2). Extract ion chromatograms are shown for matrix spiked with 50 μg L^−1^ LAi, 5 μg L^−1^ PAi, 12.5 μg L^−1^ FAi, 2.5 μg L^−1^ SAi, 5 μg L^−1^ 2OHGAi, 2.5 μg L^−1^ αKGi, and 2.5 μg L^−1^ CAi and are vertically offset for clarity

A set of calibration standards was derivatized using the protocol described in “Optimization of derivatization reagent concentration, temperature, and reaction time” to investigate the utility of the method. Limits of detection for derivatized species ranged from 0.2 to 44 μg L^−1^. These limits were generally between those reported for MPEA [20] and OBHA [21] derivatized TCA intermediates, however, comparison of these results does not account for experimental and instrumental differences. A set of experiments was conducted in porcine skeletal muscle extract to estimate matrix effects using a method described elsewhere [23] and the results suggest that this method is applicable for use in biological tissue analysis with matrix effects ranging between 94 and 107% for the species that had readily available isotope standards (Table 1). Chromatograms showing matrix alone and spiked with the lowest concentration isotope standards are shown in Fig. 5.

### Stability of Derivative

To investigate the stability and optimal storage conditions of the derivatives, a set of 50 μg L^−1^ SA standards was derivatized with 4-BNMA and stored in different common storage temperatures and then compared against freshly derivatized 50 μg L^−1^ SA d_4_ derivatized with 4-BNMA (Fig. 3). Stability was largely similar to observations in other amides [20, 21]. At room temperature (22 °C), the samples did not have significant degradation in 24 h; however, longer storage requires cooling to avoid significant degradation. 4-BNMA derivatives are stable if stored at either 4 or − 20 °C, allowing for analysis of large batches of samples.

### Applications

#### In Vitro Studies

To explore the potential applications of 4-BNMA derivatization in cell culture studies, we examined the influence of SFN on the size of the TCA intermediate pool in a cardiac cardiomyoblasts H9c2 [25] and a cancer cell model MAT BIII [26]. SFN is a phytochemical found in cruciferous vegetables that has been investigated in several chemopreventive applications [27–29]. We have recently found SFN to provide cardioprotection against doxorubicin induced cardiomyopathy and to improve mitochondrial respiratory function in vivo [30]. Cardiomyoblasts incubated with SFN exhibited increased TCA pool size while MAT BIII cancer cells incubated with SFN had decreased TCA pool size (Fig. 5).

#### Tissue Studies

**Fig. 6:**
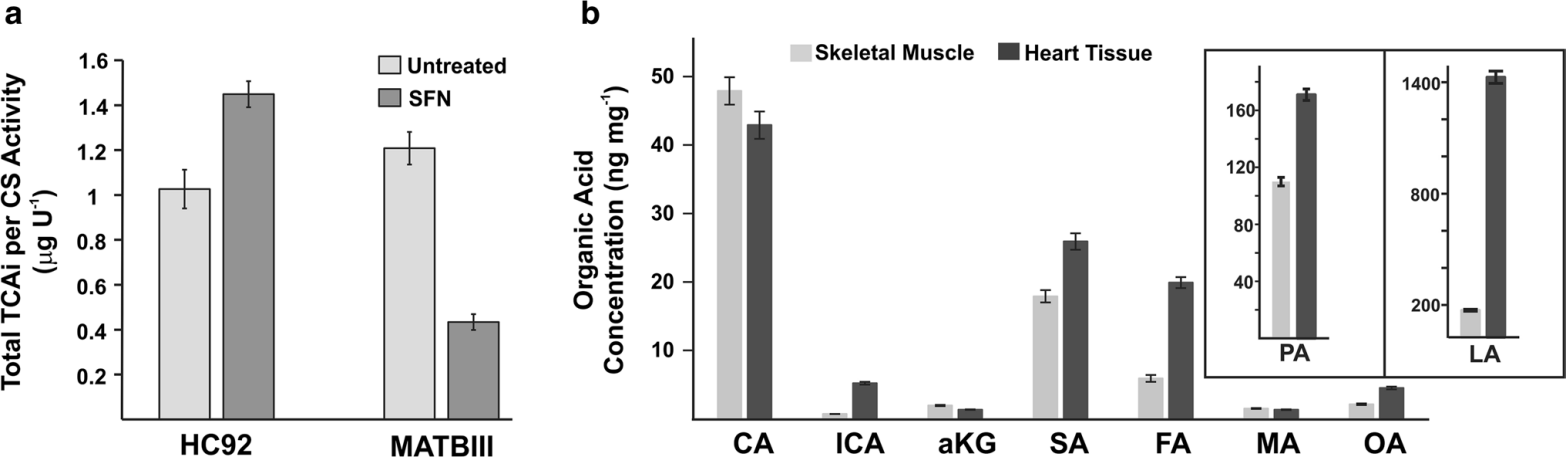
Applications of 4-BNMA derivatization technique. **a** Baseline and 10 μM treated sulforophrane (SFN) levels of total TCA normalized by citrate synthase activity for cardiomyocyte cultures (H9c2) and carcinoma cell cultures (MATBIII) as determined by 4-BNMA LC–MS/MS method. **b** Levels of biological organic acids present in porcine skeletal muscle and heart tissue as determined by 4-BNMA LC–MS/MS method

Since the TCA cycle plays a central role in the metabolic flexibility [31] of energy production in cardiac and skeletal muscle [32], we investigated the potential of the 4-BNMA method for the quantification of TCA intermediates in these tissues. Approximately, 20 g of heart tissue and skeletal muscle were collected from a recently killed 2-month-old pig for method development purposes. We tested the efficiency and reproducibility of sample processing using a bead beater type homogenizer on frozen skeletal muscle samples with different types of beads: ceramic 1.4 and 2.8 mm, 1.0 mm stainless steel, and silicon carbide beads. We found the silicon carbide beads to produce the most efficient and reproducible extracts and we found that hand grinding the tissue in a liquid N_2_ cooled mortar and pestle prior to bead beating provided no advantage (see Electronic Supplementary Material Figure SI 3). We speculate that the sharp edges of the crystalline silicon carbide beads led to more efficient tearing of the fibrous tissue. We found the most efficient extraction was conducted with MeOH–H_2_O (50:50; v/v), pre-chilling the sample at −80 °C prior to bead beating. With our optimized sample processing procedure and 4-BNMA derivatization analytical method, we found good reproducibility with average RSD of 3.5% for both tissue types (Fig. 6).

## Conclusion

The new derivatization method featuring a commercially available 4-BNMA label is applicable for an array of biologically important carboxylic acids and provides completed derivatization of multi-acid organic species such as TCA cycle intermediates. This method provides rapid analysis of biologically relevant organic acids with excellent reproducibility and with limits of detection in the low nM range for most species. The easily recognizable isotope pattern of a bromine present in the label species provides easy positive identification of derivatized species in both molecular ion and largest-product fragments. The bromine isotope pattern present in the molecular ion conveys structural information confirming the number of carboxylic acids present in the analyte providing advantages for identifying unknown species when an MS is run in precursor scan mode for the 169/171 *m*/*z* fragment in non-targeted experiments.

## Electronic supplementary material

Below is the link to the electronic supplementary material.

Supplementary material 1 (DOCX 652 kb)
