# Supplementary material for: A New Derivatization Reagent for HPLC–MS Analysis of Biological Organic Acids
Source: Chromatographia. 2017 Oct 29;80(12):1723–32. doi: 10.1007/s10337-017-3421-0 (PMC5698372; doi:10.1007/s10337-017-3421-0)
Supplement: Supplementary file 1 — Supplementary material 1 (DOCX 652 kb) [file 10337_2017_3421_MOESM1_ESM.docx]

Supplementary Information

A new derivatization reagent for high performance liquid chromatography mass spectrometry analysis of biological organic acids.

Bryce J. Marquis, Ph.D.; Hayley P. Louks; Chhanda Bose, Robert R. Wolfe and Sharda P. Singh, Ph.D.


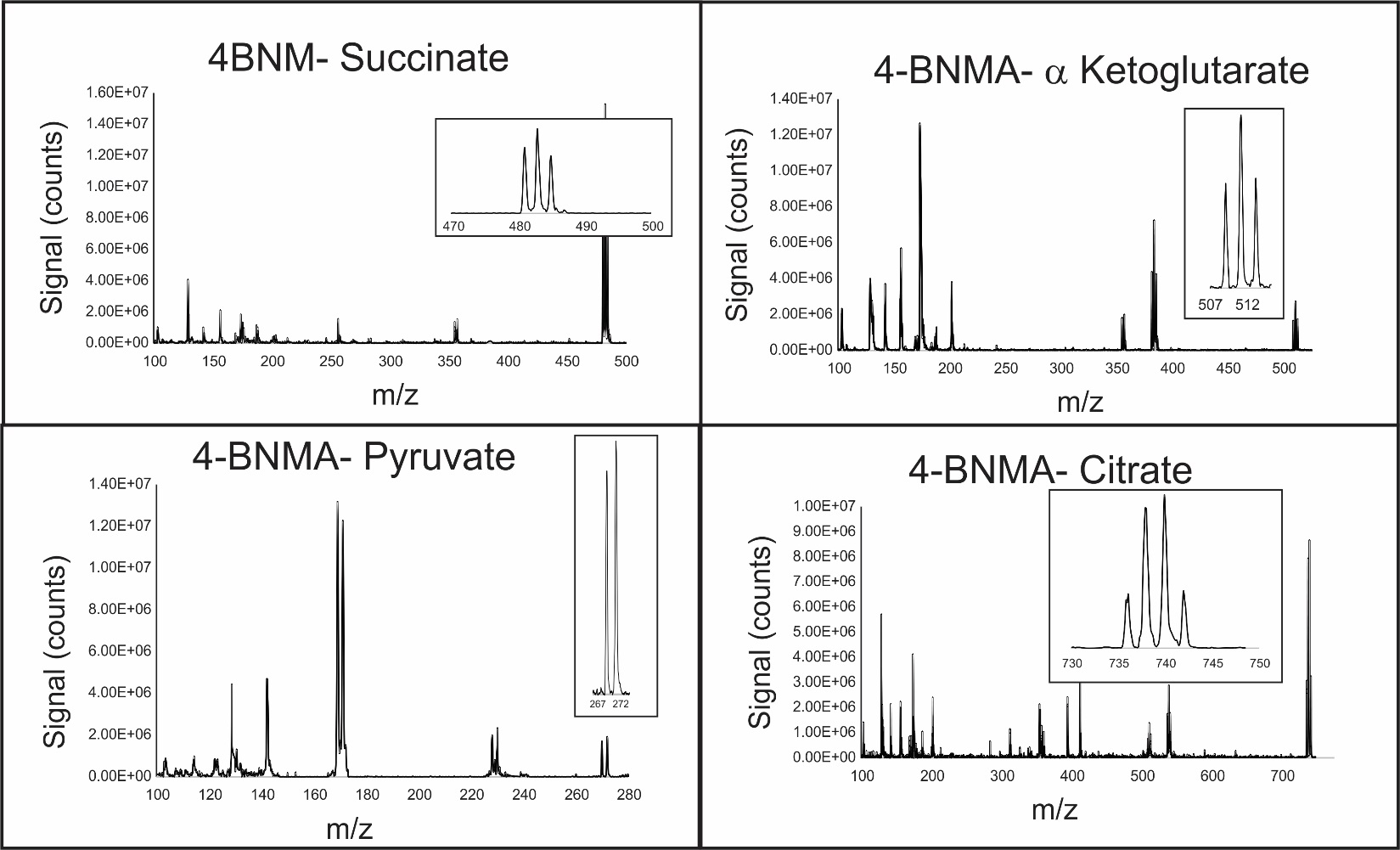


**SI1**: Example Mass spectrum from 4-BNMA derived compounds with expanded view showing precursor ion patterns for pyruvate, succinate, alpha ketoglutarate, and citrate.


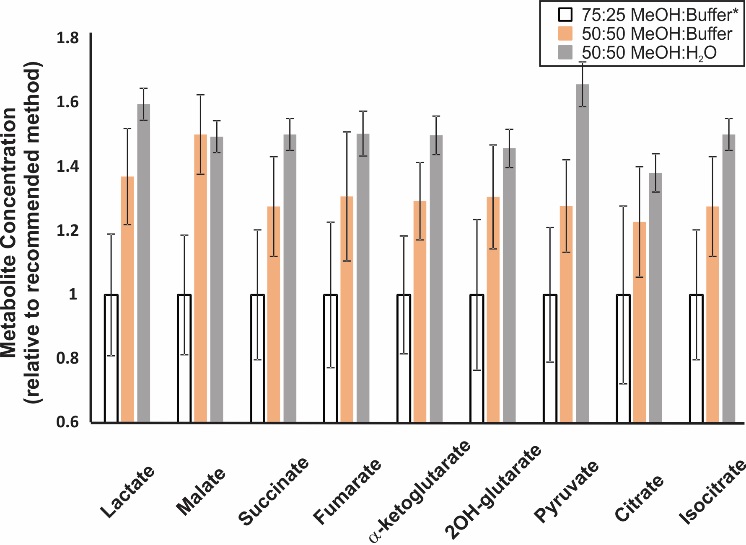


**SI 2**: Muscle sample preparation optimization of extraction buffer for 4-BNMA analysis of TCA intermediates expressed relative to 75:25 MeOH : 10 mM phosphate buffer pH 7.4 (“buffer”). Error bars represent +/- standard deviation from mean.


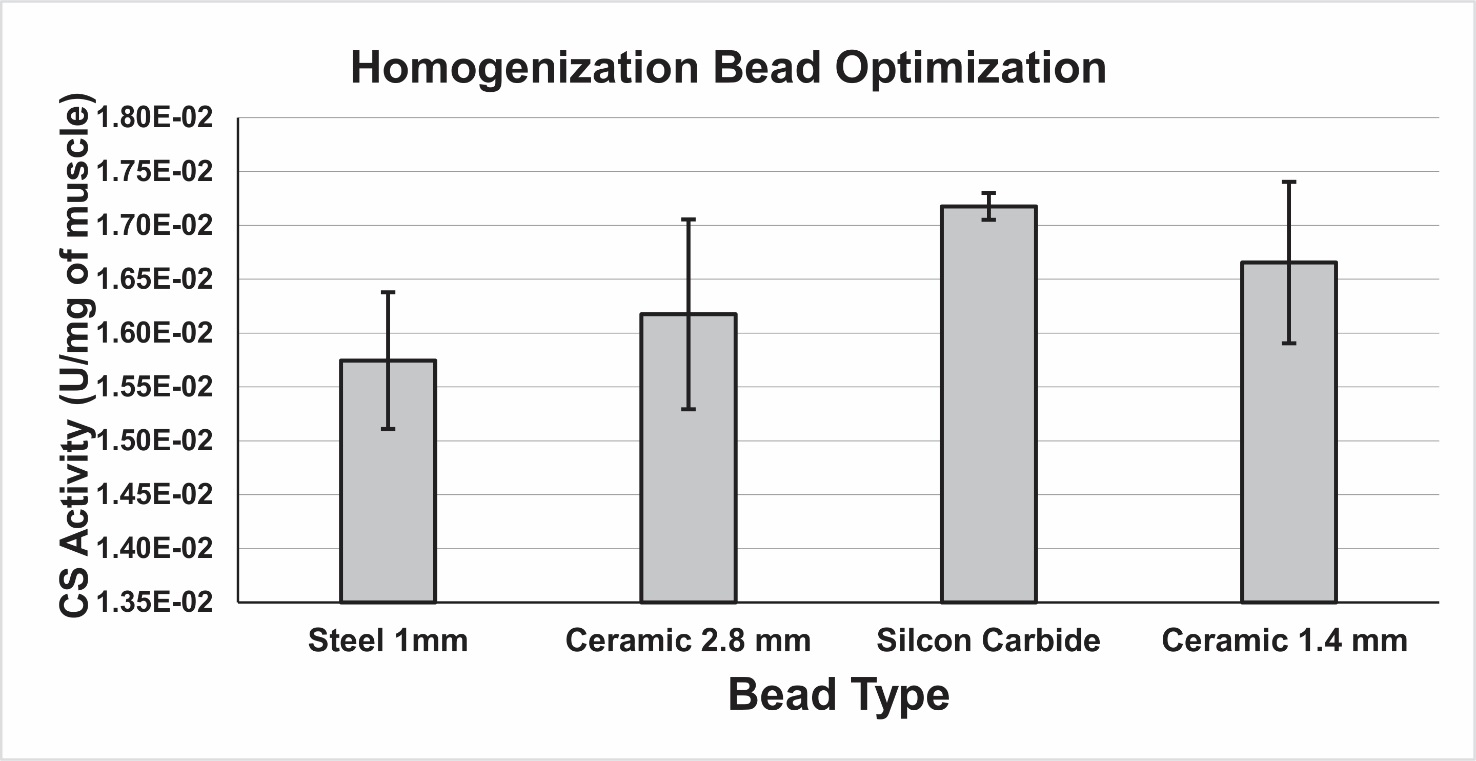


**SI 3**: Muscle sample preparation optimization of homogenization beads. Error bars represent +/- standard deviation from mean.


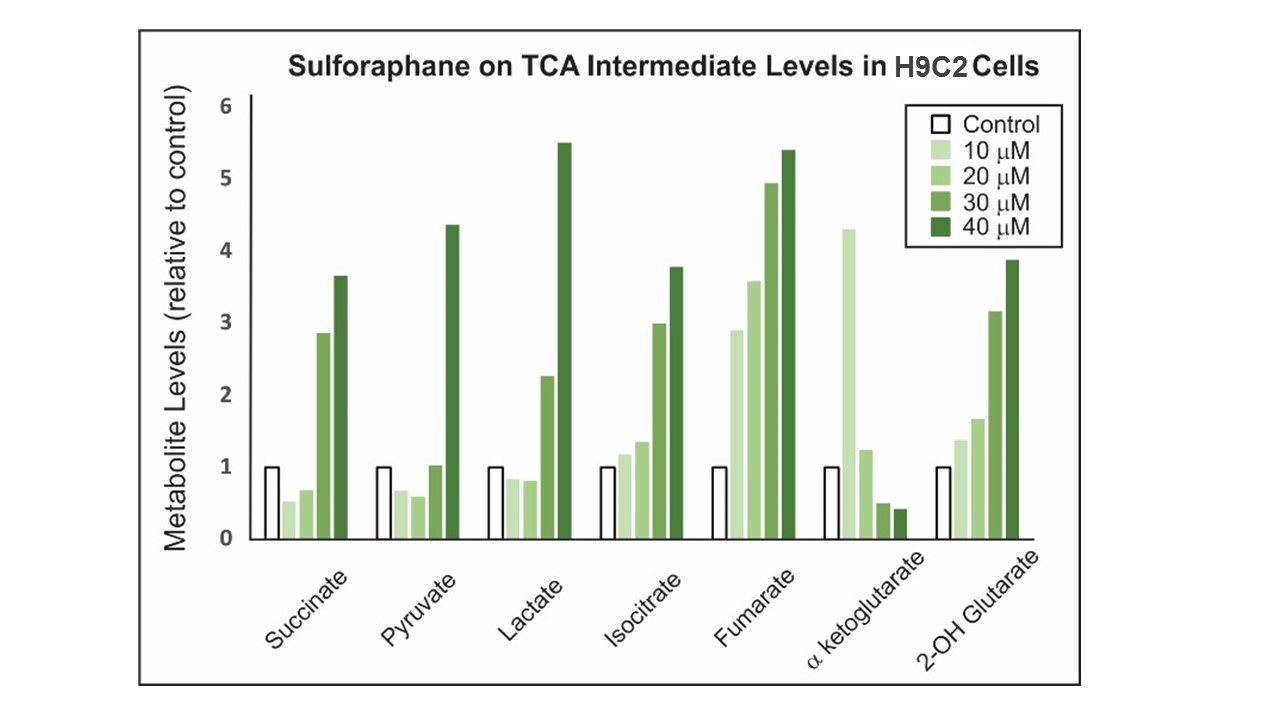


**SI 4:** Relative concentration of TCA intermediates in H9C2 cardiomyocytes after incubation with sulforphrane at different concentrations.
